# Multi-institutional comparison of treatment planning using stereotactic ablative body radiotherapy for hepatocellular carcinoma – benchmark for a prospective multi-institutional study

**DOI:** 10.1186/1748-717X-8-113

**Published:** 2013-05-04

**Authors:** Takahisa Eriguchi, Atsuya Takeda, Yohei Oku, Satoshi Ishikura, Tomoki Kimura, Shuichi Ozawa, Takeo Nakashima, Yukinori Matsuo, Mitsuhiro Nakamura, Yasuo Matsumoto, Sadanori Yamazaki, Naoko Sanuki, Yoshinori Ito

**Affiliations:** 1 Radiation Oncology Center, Ofuna Chuo Hospital, Kanagawa, Japan; 2 Department of Radiation Oncology, Juntendo University, Tokyo, Japan; 3 Department of Radiology, Hiroshima University Graduate School of Biomedical Sciences, Hiroshima, Japan; 4 Department of Radiation Oncology and Image-applied Therapy, Kyoto University, Kyoto, Japan; 5 Department of Radiation Oncology, Niigata Cancer Center Hospital, Niigata, Japan; 6 Department of Radiation Oncology, National Cancer Center Hospital, Tokyo, Japan

**Keywords:** Stereotactic body radiotherapy, Stereotactic ablative body radiotherapy, SBRT, SABR, Benchmark, Clinical study, Hepatocellular carcinoma

## Abstract

**Introduction:**

Several single institution phase I and phase II trials of stereotactic ablative body radiotherapy (SABR) for liver tumors have reported promising results and high local control rates of over 90%. However, there are wide variations in dose and fractionation due to different prescription policies and treatment methods across SABR series that have been published to date.

This study aims to assess and minimize inter-institutional variations in treatment planning using SABR for hepatocellular carcinoma (HCC) in preparation for a prospective multi-institutional study.

**Methods:**

Four institutions (A-D) participated in this study. Each institution was provided with data from four cases, including planning and diagnostic CT images and clinical information, and asked to implement three plans (a practice plan and protocol plans 1 and 2). Practice plans were established based on the current treatment protocols at each institution. In protocol plan 1, each institution was instructed to prescribe 40 Gy in five fractions within 95% of the planning target volume (PTV). After protocol plan 1 was evaluated, we made protocol plan 2, The additional regulation to protocol plan 1 was that 40 Gy in five fractions was prescribed to a 70% isodose line of the global maximum dose within the PTV. Planning methods and dose volume histograms (DVHs) including the median PTV D50 (D_m_50) and the median normal liver volume that received 20 Gy or higher (V_m_20) were compared.

**Results:**

In the practice plan, D_m_50 was 48.4 Gy (range, 43.6-51.2 Gy). V_m_20 was 15.9% (range, 12.2-18.9%). In protocol plan 1, the D_m_50 at institution A was higher (51.2 Gy) than the other institutions (42.0-42.2 Gy) due to differences in dose specifications. In protocol plan 2, variations in DVHs were reduced. The D_m_50 was 51.9 Gy (range, 51.0-53.1 Gy), and the V_m_20 was 12.3% (range, 10.4-13.2%). The homogeneity index was nearly equivalent at all institutions.

**Conclusions:**

There were notable inter-institutional differences in practice planning using SABR to treat HCC. The range of PTV and normal liver DVH values was reduced when the dose was prescribed to an isodose line within the PTV. In multi-institutional studies, detailed dose specifications based on collaboration are necessary.

## Introduction

Hepatocellular carcinoma (HCC) primarily affects patients with chronic liver disease. Patients with chronic hepatitis or cirrhosis secondary to viral hepatitis B or C and alcoholism are at the highest risk of developing HCC. Clinical practice guidelines [1,2] recommend surgical resection, transplantation or percutaneous ablation to treat solitary HCC in patients with adequate liver function.

Stereotactic ablative body radiotherapy (SABR) is an emerging treatment modality that enables delivery of ablative doses to tumors with acceptable toxicity. Several single institution phase I and phase II trials of SABR for liver tumors have reported promising results and high local control rates of over 90% [3-6]. Additional multi-institutional prospective studies could establish this as an alternative treatment for patients who are ineligible for other local treatments for solitary HCC. However, there are wide variations in dose and fractionation due to different prescription policies and treatment methods across SABR series that have been published to date [3,4,7-9].

We assessed inter-institutional variations in SABR planning to treat HCC and run a benchmark in preparation for a multi-institutional prospective study.

## Methods

### Study schemes

**Figure 1:**
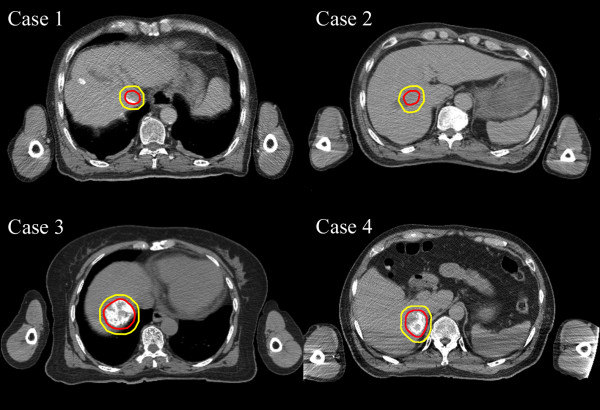
**Planning computed tomography images.** Outer and inner lines indicate the planning target volume and gross tumor volume, respectively. Case 1, hepatocellular carcinoma (23 mm) located in segment 1 (S1) near the duodenum; case 2, hepatocellular carcinoma (25 mm) located just below the diaphragm in S4; case 3, hepatocellular carcinoma (22 mm) located in S5 near the inferior vena cava and the duodenum; and case 4, hepatocellular carcinoma (40 mm) located in S6/7.

Four institutions (A, B, C and D) participated in this study. Anonymized data from four benchmark cases with HCC were distributed to the participating institutions, including planning computed tomography (CT) images, pretreatment triphasic CT images and clinical information. Planning CT images from each case are shown in Figure 1. The tumors were in different locations with maximum tumor diameters of 22, 23, 25 and 40 mm. Structure sets of the liver, gross tumor volume (GTV), internal target volume (ITV) and planning target volume (PTV) were also provided. The PTV of case 1, 2, 3, and 4 were 35.4, 50.5, 87.6 and 105.8 cc, respectively. Pretreatment triphasic CT images were acquired at a resting expiratory level with the patient in a vacuum pillow and under abdominal compression. Planning CT images were acquired in a long scan (6–8 seconds/slice) during free breathing. GTV was contoured on pretreatment triphasic CT and combined with planning CT. CTV was equated to GTV. ITV was inserted on the planning CT image by adding margins (2–6 mm) to the GTV according to respiratory movements measured by fluoroscopy. PTV was determined by adding 2 mm to the ITV. Normal liver was defined as the liver minus GTV. The four patients were informed regarding use of their clinical data for this study and provided written informed consent.

### Treatment plans

At each institution, planning CT images and structure sets were imported into a radiotherapy treatment planning system (TPS), and study plans were created. The beam x-ray energy was set at 6 MV. Different dose calculation algorithms were allowed.

Routine clinical plans (practice plans) were established according to current treatment protocols at each institution, including prescription dose, prescription point and dose constraints. Another plan included prescribing 40 Gy in five fractions at 95% of the PTV (protocol plan 1). After analyzing and discussing the results of protocol plan 1, each institution was asked to implement an additional plan in which 40 Gy in five fractions was prescribed at the 70% isodose line of the global maximum dose within the PTV in which 95% of the PTV received more than 40 Gy (protocol plan 2).

### Plan comparisons

Planning CT images, structure sets, plans and doses from each institution were collected and imported to the treatment planning system (Eclipse, version 10.0, Varian, Palo Alto, CA). The following items were also collected and compared: radiotherapy unit, radiotherapy TPS, dose calculation algorithm, prescription dose, prescription point, beam arrangement, planning CT methods, target volume delineation methods and dose constraints. Dose volume histograms (DVHs) of the GTV, PTV and normal liver from each plan at each institution were evaluated. Median D50 (D_m_50), D_m_90, D_m_98, maximum dose and minimum dose were acquired. Median normal liver volume receiving 20 Gy or higher (V_m_20) and median mean normal liver dose (MLD_m_) were used to evaluate the normal liver dose. For GTV and PTV, the homogeneity index (HI) was defined as the maximum dose delivered to 2% of the target volume (D2) minus D98 divided by D50. Dose conformity was evaluated in terms of conformation number (CN) [10], quantified as:

$$\left(V_{T,\mathrm{pi}}/V_{T}\right)*\left(V_{T,\mathrm{pi}}/V_{\mathrm{pi}}\right),$$

where: V_T, pi_ = volume within the PTV receiving a dose ≥ the prescription dose, V_pi_ = volume receiving a dose ≥ the prescription dose, V_T_ = PTV. We had defined metrics for planning evaluation before protocol plan 1, therefore we used same metrics to evaluate protocol plan 1 and 2.

## Results

Current planning and treatment protocols at participating institutions are shown in Table 1. Remarkable variations between each institution were observed. Institutions A and B used non-coplanar and coplanar dynamic conformal arc beams, respectively. In contrast, institutions C and D used non-coplanar static beams. At institution A, the prescribed dose was to the 70% isodose line within the PTV surface, while the other three institutions prescribed to an isocenter.

**Table 1:** Planning and treatment parameters

	**A**	**B**	**C**	**D**
TPS	XiO	iPlan	iPlan	Pinnacle
Calculation algorithm	SuperPosition	Pencil Beam	XVMC	CCCS
Prescription dose	ChildA: 40Gy/5fr	44Gy/4fr	Peripheral: 48Gy/4fr	Peripheral: 48Gy/4fr
	ChildB: 35Gy/5fr		PeriGI:60Gy/8fr	Cenrtral:60Gy/8fr
Prescription point	70% isodose	Isocenter	Isocenter	Isocenter
Beam arrangement	Dynamic conformal arc	Dynamic conformal arc	Static	Static
	Non-coplanar, 8 arcs	Coplanar, 2 arcs	Non-coplanar, 6–8 beams	Non-coplanar, 6-8 arcs
Respiratory movement	Confined free-breathing(<1cm)	Confined free-breathing	Confined free-breathing	Gated by Abches® or RPM
	Under abdominal compression	Under abdominal compression	Under abdominal compression	
Target definition CT	Fusion of LSTCT + dynamic CT	Fusion of 4 times breathing CT	Fusion of 4DCT + breath-hold CT	Breath-hold CT
Planning CT	LSTCT	LSTCT	AveIP from 4DCT	Breath-hold CT
ITV	GTV + 2-6mm	Fusion of 4 times breathing CT	Directly visualized by LSTCT	ITV = GTV
PTV	ITV + 2mm	ITV + CC10mm, AP, LR8mm	ITV + 5mm	ITV + CC8mm, AP, LR5mm
Dose constraints				
Liver	V_20_(include PTV) < 20%	V_<16_ > 700 cc	V_20_ < 25% V_<15_ > 700 cc	V_20_≦25%
Spinal cord	D_max_ < 25Gy, D_1cc_ < 20Gy	D_max_ < 25Gy/4fr	D_max_ < 25Gy/4fr	D_max_ < 25Gy/4fr
GI tract	D_max_ < 25Gy, D_1cc_ < 20Gy	D_10cc_ < 35Gy/4fr	D_1cc_ < 40Gy/4fr, D_10cc_ < 35Gy/4fr	D_1cc_ < 30Gy/4fr, D_10cc_ < 24Gy/4fr
Kidney	-	V_20_ < 30%	V_20_ < 30%	D_1/3_ < 20Gy/4fr
Heart	-	-	-	D_1/3_ < 28Gy/4fr
Gallbladder	-	-	-	D_1/3_ < 40Gy/4fr

Abbreviations: *TPS*, treatment planning system; *XVMC*, X-ray voxel Monte Carlo algorithm; *CCCS*, collapsed cone convolution superposition; *periGI*, perigastrointestinal; *RPM*, The Varian® Real-time Position Management™ system; *GI*, gastrointestinal; *LSTCT*, long scan time computed tomography; *4DCT*, 4-dimensional computed tomography; *AveIP*, average intensity projection; *GTV*, gross tumor volume; *PTV*, planning target volume; *ITV*, internal target volume; *CC*, cranial and caudal; *LRA*, left, right and anterior; *P*, posterior; *LR*, left and right; *AP*, anterior and posterior.

### Practice plan

**Figure 2:**
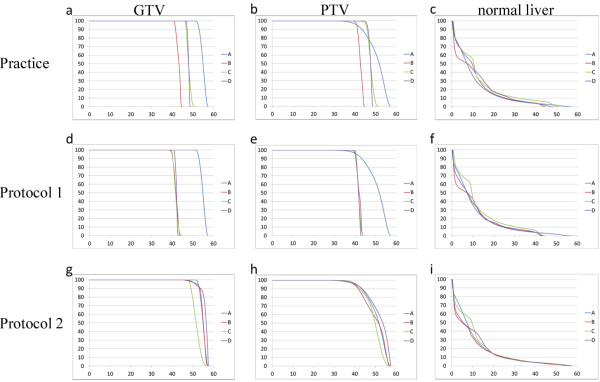
**Dose volume histograms of case 4.** (**a**) GTV, (**b**) PTV and (**c**) normal liver in the practice plan; (**d**) GTV, (**e**) PTV and (**f**) normal liver in protocol plan 1; and (**g**) GTV, (**h**) PTV and (**i**) normal liver in protocol plan 2.

**Figure 3:**
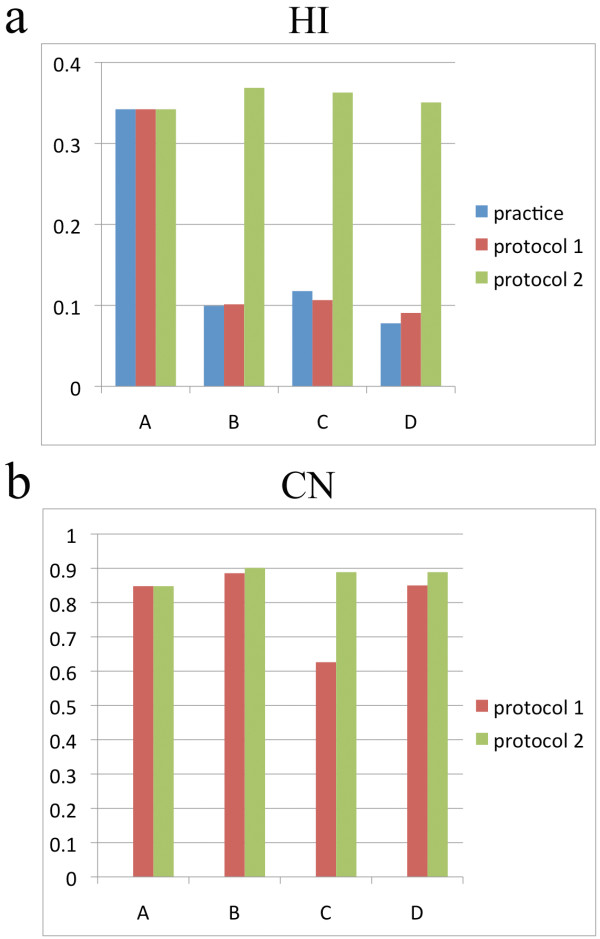
**Homogeneity index (HI) and conformation number (CN) at institutions A-D.** Median (**a**) HI and (**b**) CN at institutions A-D.

GTV, PTV and normal liver DVHs for case 4 are shown in Figure 2a-c. The shoulder of the PTV DVH had a lower gradient and higher maximum dose at institution A compared to the other institutions. There was significant variation among DVHs in target volume, although low variation was observed in the normal liver. D_m_95 and D_m_50 of PTV of the four cases were 43.5 Gy (range, 41.1-46.5 Gy) and 48.4 Gy (range, 43.6-51.2 Gy), respectively. V_m_20 and MLD_m_ were 15.9% (range, 12.2-18.9%) and 10.8% (range, 8.8-12.5%), respectively. Median HI was higher at institution A (Table 2, Figure 3).

**Table 2:** Dose-volumetric data of target volumes

			**A**	**B**	**C**	**D**
				**Median (Min - Max)**		
Practice-plan						
GTV	D_m_50	(Gy)	55.2(52.6-55.8)	44.1(43.3-44.2)	49.0(44.2-55.5)	47.9 (46.6 - 48.5)
	D_m_95	(Gy)	53.1(48.8-54.2)	42.7(41.3-43.7)	47.6(44.2-54.3)	47.2 (46.6 - 47.8)
	D_m_98	(Gy)	52.3(47.8-53.8)	42.6(41.1-43.6)	47.5(43.9-54.0)	47.2 (46.4 - 47.7)
	Min	(Gy)	50.0(49.3-53.1)	42.2(40.9-43.3)	46.8(43.1-53.4)	46.3 (39.6 - 47.0)
	Max	(Gy)	57.1(57.1-57.1)	45.1(44.6-47.8)	51.8(50.9-57.2)	49.0 (41.2 - 49.7)
PTV	D_m_50	(Gy)	51.2(49.5-52.1)	43.6(42.7-44.0)	49.0(46.4-55.4)	47.7 (47.5 - 47.8)
	D_m_95	(Gy)	41.2(41.0-41.5)	41.1(40.7-41.9)	46.5(43.2-53.4)	45.7 (45.5 - 46.5)
	D_m_98	(Gy)	39.0(38.4-39.8)	40.5(39.8-41.3)	45.9(42.5-52.9)	45.3 (44.7 - 46.1)
	Min	(Gy)	32.5(27.5-34.0)	37.1(35.4-37.3)	40.5(37.7-50.7)	43.7 (41.9 - 45.0)
	Max	(Gy)	57.1(57.1-57.1)	45.2(44.7-48.9)	40.5(51.7-57.7)	43.7 (48.7 - 49.9)
HI			0.34(0.33-0.36)	0.10(0.08-0.19)	0.12(0.08-0.18)	0.08 (0.06 - 0.09)
V_m_20 (normal liver)		(%)	13.2(6.7-14.1)	12.2(6.5-14.9)	18.9(13.7-22.3)	18.5 (10.1 - 23.2)
MLD_m_		(Gy)	9.3(7.1-11.2)	8.8(7.1-10.4)	12.5(10.9-13.2)	12.2 (8.7 - 14.0)
Protocol-plan1						
GTV	D_m_50	(Gy)	55.2(52.6-55.8)	42.7(42.0-43.1)	42.0(41.6-43.1)	42.4 (42.2 - 42.7)
	D_m_95	(Gy)	53.1(48.8-54.2)	41.6(40.2-42.0)	40.8(40.6-41.2)	44.5 (41.2 - 42.7)
	D_m_98	(Gy)	52.3(47.8-53.8)	41.5(40.0-42.0)	40.6(40.4-41.0)	41.4 (41.1 - 42.1)
	Min	(Gy)	50.0(49.0-53.0)	41.0(39.7-42.0)	39.9(39.6-40.4)	41.0 (40.7 - 42.0)
	Max	(Gy)	57.1(57.1-57.1)	57.1(43.1-46.7)	50.6(43.1-47.6)	45.4 (43.1 - 44.5)
PTV	D_m_50	(Gy)	51.2(49.5-52.1)	42.2(41.8-43.1)	42.0(41.6-43.1)	42.0 (41.6 - 42.2)
	D_m_95	(Gy)	41.2(41.0-41.5)	40.0(40.0-40.1)	40.1(40.1-40.2)	40.0 (40.0 - 40.2)
	D_m_98	(Gy)	39.0(38.4-39.8)	39.5(39.4-39.6)	39.7(39.7-39.8)	39.6 (39.2 - 39.8)
	Min	(Gy)	32.5(27.5-34.0)	36.4(34.1-37.5)	38.9(38.6-39.1)	37.3 (36.6 - 40.7)
	Max	(Gy)	57.1(57.1-57.1)	44.2(43.3-48.2)	45.1(43.3-48.8)	43.5 (43.4 - 44.5)
HI			0.34(0.33-0.36)	0.10(0.09-0.19)	0.11(0.07-0.17)	0.09 (0.07 - 0.10)
CN			0.85(0.81-0.91)	0.89(0.80-0.90)	0.63(0.59-0.68)	0.85(0.75 - 0.86)
V_m_20 (normal liver)		(%)	13.2(6.7-14.1)	11.7(6.2-14.6)	17.5(10.0-19.1)	14.6(8.8 - 15.5)
MLD_m_		(Gy)	9.3(7.1-11.2)	8.8(6.9-9.9)	11.4(8.6-12.3)	10.2 (7.6 - 10.4)
Protocol-plan2						
GTV	D_m_50	(Gy)	55.2(52.6-55.8)	55.3(53.2-56.3)	53.6(51.6-55.0)	56.2 (55.9 - 56.6)
	D_m_95	(Gy)	53.1(48.8-54.2)	53.2(50.7-55.6)	50.6(48.6-53.3)	52.6 (51.2 - 54.7)
	D_m_98	(Gy)	52.3(47.8-53.8)	52.2(47.4-55.3)	50.1(48.0-52.8)	50.3 (47.4 - 53.0)
	Min	(Gy)	50.0(49.3-53.1)	51.5(45.0-54.7)	49.7(47.1-53.8)	47.0 (42.6 - 50.5)
	Max	(Gy)	57.1(57.1-57.1)	57.1(57.1-57.1)	57.1(57.1-57.1)	57.1 (57.1 - 57.1)
PTV	D_m_50	(Gy)	51.2(49.5-52.1)	52.5(51.5-54.0)	51.0(49.9-52.3)	53.1 (50.8 - 53.8)
	D_m_95	(Gy)	41.2(41.0-41.5)	40.0(40.0-40.2)	40.1(40.0-40.3)	40.6 (40.4 - 41.3)
	D_m_98	(Gy)	39.0(38.4-39.8)	37.1(36.7-37.7)	37.8(37.4-38.2)	38.5 (36.3 - 38.9)
	Min	(Gy)	32.5(27.5-34.0)	21.5(18.5-23.6)	26.9(24.9-28.8)	29.5 (25.8 - 32.4)
	Max	(Gy)	57.1(57.1-57.1)	57.1(57.1-57.1)	57.1(57.1-57.1)	57.1 (57.1 -57.1)
HI			0.34(0.33-0.36)	0.37(0.36-0.38)	0.36(0.35-0.39)	0.35 (0.35 -0.39)
CN			0.85(0.81-0.91)	0.90(0.88-0.91)	0.89(0.87-0.91)	0.89 (0.85 -0.89)
V_m_20 (normal liver)		(%)	13.2(6.7-14.1)	11.5(6.3-14.0)	10.4(6.3-13.7)	13.0 (6.8 -13.7)
MLD_m_		(Gy)	9.3(7.1-11.2)	8.3(7.0-9.9)	8.9(8.0-10.9)	9.5 (6.4 - 10.2)

Abbreviations: *GTV*, gross tumor volume; *PTV*, planning target volume; *HI*, homogeneity index; *CN*, conformation number; *MLD*, mean liver dose.

### Protocol plan 1

In protocol plan 1 (Table 2, Figure 2d-f), all of the institutions used a D95 of 40 Gy in all cases. Except for GTV and PTV DVHs at institution A, there was little variation in GTV, PTV and normal liver DVHs at each institution. PTV D_m_50 at institution A was higher than at the other institutions. V_m_20 and MLD_m_ were 13.9% (range, 11.7-17.5%) and 9.8 Gy (range, 8.8-11.4 Gy), respectively. HI was higher at institution A (Table 2, Figure 3). CN at institution C was relatively lower than at the other three institutions.

### Protocol plan 2

**Figure 4:**
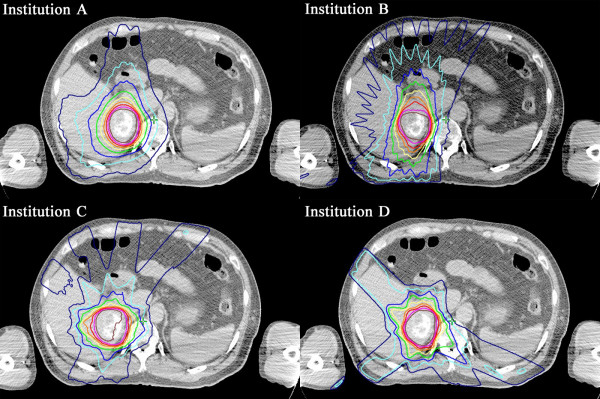
Dose distribution for case 4 in protocol plan 2.

In protocol plan 2 (Table 2, Figure 2g-i), all of the institutions complied with the dose constraints. Regarding variation among GTV and PTV DVHs in protocol plan 2, the range of DVH values was reduced compared with protocol plan 1. Although the DVH shape was similar to the shape observed in the practice plan and protocol plan 1, V_m_20 and MLD_m_ were lower than the other two plans. Dose distribution at each institution is shown in Figure 4. Median HI and CN values were nearly equivalent at all institutions (Table 2, Figure 3).

## Discussion

SABR is expected to be a treatment option indicated for HCC patients who are ineligible for surgery or radiofrequency ablation. However, various dose prescription and treatment planning strategies are currently used by different groups [3,4,7-9] and an optimal dose has not been determined.

For trials involving advanced radiation therapy techniques, the minimum acceptable degree of protocol compliance must be described to mitigate unacceptable variation between institutions [11]. This study revealed many differences in planning and treatment protocols at several institutions (Table 1). In conducting a clinical trial of SABR, treatment planning can vary based on multiple factors, such as planning CT, target volume delineation, beam arrangement, dose calculation algorithms and prescription point [12]. It is difficult to unify the method to acquire planning CT because treatment modalities vary among institutions. In regard to measures to account for respiratory movement, it is important to set up some criteria with acceptable range in preparing for a protocol. Calculation algorithms have influence on dose distribution when some beams pass through materials with air density, therefore newer generation calculation algorithms such as superposition or comparable algorithm may be preferable. Variations in target delineation have been reported by several investigators [13,14]. Delineation of HCC can also be affected by scanning protocol of triphasic CT, with or without use of MRI. In this study, identical target volumes were intentionally delineated prior to data distribution to eliminate variation and enable direct comparison of DVH parameters used in different planning methods.

In the practice plan, PTV dose distribution varied among institutions due to differences in prescription dose and prescription point. A uniform prescription dose of 40 Gy in five fractions administered as D95 were required in protocol plan 1. As a result, there was a significant gap between institution A and the other three institutions (Figure 2d-f) due to different prescription methods because institution A prescribed at the 70% isodose level relative to the global maximum dose, while the other three institutions prescribed at the isocenter.

There are two different concepts regarding dose within the target in SABR. One maintains dose homogeneity within the target, which is generally prescribed at the isocenter. The concept has been widely utilized in Japan. In the other concept, dose is prescribed at the PTV margin and does not maintain dose homogeneity [15]. In the latter concept, there is another variation in prescription method which provides more flexibility and is more treatment planning system and technique independent. In a randomized phase III trial of Radiosurgery Or Surgery for operable Early stage (stage 1A) non-small cell Lung cancer (ROSEL) study, the dose prescription was based on D95 of the PTV receiving at least the nominal fraction dose, and D99 of the PTV receiving a minimum of 90% of the fraction dose. The dose maximum within the PTV should preferably be between 110% and 140% of the prescribed dose. The location of the treatment plan normalization point can be left to the institutions preference [16].

In conventional radiotherapy, International Commission on Radiation Units and Measurements (ICRU) Report 50 [17] recommends a uniform dose to the target volume within −5% to +7% of the prescribed dose with a radiation dose at the reference point, which is generally the isocenter. In contrast, dose heterogeneity within the target is acceptable in SABR for targets that do not involve functional normal tissue, as outlined in best practice guidelines by the American Association of Physicists in Medicine (AAPM) Task Group 101 [18]. By ignoring dose homogeneity within the PTV, tight conformity with steep and isotropic dose fall-off and high dose delivery to the target volume can be achieved in addition to a simultaneous reduction in the normal tissue dose [19]. In this study, institution A prescribed the dose at a 70% isodose line. Accordingly, protocol plan 2 required dosing to the 70% isodose line of the global maximum dose within 95% of the PTV. As a result, GTV and PTV doses were increased in protocol plan 2, while the normal liver dose decreased compared with protocol plan 1.

Improvements in DVH were primarily attributed to prescribing the dose at the 70% isodose line. Widder et al. [20] reported that dose prescription in SABR for lung cancer at isodose levels between 50% and 70% of the dose at the isocenter resulted in a lower dose to surrounding tissues and lungs compared with an 80% isodose level. Although there are no reports on optimal isodose levels for SABR to treat HCC, prescription to the 70% isodose level rather than an isocenter improved dose distribution in the current study.

Differences in DVH parameters between institutions, particularly in the V20 and MLD in the practice plan and protocol plan 1, were grouped according to static and dynamic beam arrangements. Institutions A and B, which used a dynamic conformal arc, had lower V20 and MLD values than institutions C and D, which used non-coplanar static beams. Although a greater number of beams generally results in better conformity and dose distribution gradients, six to eight non-coplanar static beams sufficiently fulfilled the planning requirement in protocol plan 2. Prescription at the 70% isodose line successfully reduced the dose to surrounding normal tissues regardless of different beam arrangements.

In addition to improving planning quality, the current study shared treatment strategies at various institutions. After data collection, researchers from the institutions discussed their treatment planning policies and compared study results. With respect to dose distribution at each institution (Figure 4), institution C selected beam directions that increased non-irradiated normal liver volume as much as possible, while institutions A and B were not as concerned about low doses to the normal liver. Institution D indicated that avoiding as much of the gastrointestinal tract as possible rather than dose reduction in the normal liver was important. Multi-leaf collimator margin size also varied among institutions, from uniform margins around the PTV (generally 5 to 10 mm) to variable margins in three-dimensional directions, due to different dose prescription policies. This information, which was discussed in person, can favorably influence researchers toward improved treatment planning. This study uncovered possible variations in SABR planning among participating institutions and would help to prepare for a comprehensive protocol as well as to define credentialing and evaluation criteria beforehand. In multi-center clinical trials, maintaining protocol treatment quality by minimizing these variations is a challenge. Therefore, this type of study prior to establishing a protocol is in agreement with the goals of quality assurance (QA) programs that attempt to minimize variations. According to a meta-analysis and a systematic review, radiation therapy protocol deviations are associated with increased risk of treatment failure and overall mortality [21,22]. Well-organized QA programs will result in improved reliability of clinical trials and quality of practice [23].

As limitations, the current study only compared treatment planning methods directly related to SABR and did not consider other factors that could affect treatment, such as methods of planning CT acquisition, contouring of at-risk targets and organs, patient fixation and respiratory gating. Calculation algorithms were not a key focus, which could influence dose distribution under specific conditions. The impact of variations in calculation algorithms based on dose distribution should be further evaluated.

## Conclusion

In planning SABR to treat HCC, there were notable inter-institutional differences. When the dose was prescribed to an isodose line fitted to the PTV surface, prescription requirements were fulfilled and differences in DVH between institutions decreased significantly. In multi-institutional studies, detailed dose specifications based on collaboration are necessary. A thoroughly described protocol with a radiotherapy QA program will lead to high-quality treatment and reliable results.

## Competing interests

AT received grant from Varian Research Collaboration Program. SI received fees from Elekta K.K. for consultancy for the company.

The other authors declare that they have no competing interests.

## Authors’ contributions

TE analyzed data, drafted and revised the manuscript. AT, NS, SI, TK, YM, YM, YI revised the manuscript critically. YO prepared patient data sets for this planning study, collected and analyzed data. AT, YO, TK, SO, TN, YM, MN, YM, SY performed treatment planning. All the authors participated in this study design and discussion all along this study, read and approved the final manuscript.
